# Biotemplating pores with size and shape diversity for Li-oxygen Battery Cathodes

**DOI:** 10.1038/srep45919

**Published:** 2017-04-04

**Authors:** Dahyun Oh, Çagla Ozgit-Akgun, Esin Akca, Leslie E. Thompson, Loza F. Tadesse, Ho-Cheol Kim, Gökhan Demirci, Robert D. Miller, Hareem Maune

**Affiliations:** 1 IBM Almaden Research Center, San Jose, CA 95120, USA; 2 ASELSAN Inc. – Microelectronics, Guidance and Electro-Optics Business Sector, Ankara 06750, Turkey; 3 Minnesota State University Moorhead, Chemistry Department, Moorhead, MN 56563, USA

## Abstract

Synthetic porogens provide an easy way to create porous structures, but their usage is limited due to synthetic difficulties, process complexities and prohibitive costs. Here we investigate the use of bacteria, sustainable and naturally abundant materials, as a pore template. The bacteria require no chemical synthesis, come in variable sizes and shapes, degrade easier and are approximately a million times cheaper than conventional porogens. We fabricate free standing porous multiwalled carbon nanotube (MWCNT) films using cultured, harmless bacteria as porogens, and demonstrate substantial Li-oxygen battery performance improvement by porosity control. Pore volume as well as shape in the cathodes were easily tuned to improve oxygen evolution efficiency by 30% and double the full discharge capacity in repeated cycles compared to the compact MWCNT electrode films. The interconnected pores produced by the templates greatly improve the accessibility of reactants allowing the achievement of 4,942 W/kg (8,649 Wh/kg) at 2 A/g_e_ (1.7 mA/cm^2^).

Porous structures are essential for a variety of device applications. Reactant transport through a solid medium is highly dependent on matrix porosity, therefore interconnected porous structures can improve the reactant accessibility to internal reaction sites in large scale devices. For practical applications such as sensors^1^,^2^, gas storage^3^,^4^, membranes^5^, solar cells^6^ and batteries^7^, porous materials are highly desirable as they can directly enhance the device performance by improving the access of liquids or gases. Therefore, building better porous structures is of a critical importance for next generation systems because it can improve device efficiency dramatically. A common approach to generating pores in matrix materials is the direct templating method^8^, which provides an easy and controllable route for introducing homogeneous pores with diameters ranging in size from meso (2–50 nm) to macroscale (>50 nm)^9^. However, conventional pore templates; such as silica^10^, polymer beads (e.g., latex)^11^ or anodic aluminum oxide^12^ are generally expensive, complex to produce and can be difficult to completely remove. These issues often limit practical device fabrication and mass production. One such application for porous materials would be in advanced batteries such as lithium-oxygen, where the solid/gas interface of the cathode is critical. Here we propose using microorganisms (e.g., *Escherichia coli; E. coli* or *Staphylococcus epidermidis; S. epi*) as a self-regulating pore templating materials. We calculate that this approach could reduce the cost of pore template materials by ~million, 1,000,000 times for fabricating free-standing porous films relative to that of conventional pore templates. In this study, we have demonstrated the use of biomaterial templates for generating porous 3D MWCNT films and show their performance effectiveness as free-standing, binder-free cathodes for Li-oxygen batteries. These microorganisms were simple and versatile to template pores to form porous cathode films which improves the battery performance significantly.

The Li-oxygen battery is a promising, next generation energy storage system that provides a theoretical energy density three times higher than that of current Li-ion batteries^13^. During the Li-oxygen battery cycle, solid discharge products (Li_2_O_2_) are electrochemically deposited and subsequently decomposed upon charging on the cathode surface (2Li^+^ + O_2_ + 2e^−^ ↔ Li_2_O_2_). The dimensions and shapes of these solid discharge products range from thin films (a few nanometers thick, ~9 nm) to larger toroidal-shaped particles (200–500 nm), depending on the battery operation conditions (e.g. current density, capacity and electrolytes)^14^,^15^. These solid products can potentially block even macropores. In addition, because of the poor electronic conductivity of Li_2_O_2_ (5 × 10^−20^ S/cm, calculated^16^ or 10^−12^–10^−11^ S/cm at 100 °C, measured^17^), discharge products on the cathode may hinder charge transport throughout the cathode. It is important, therefore, to custom design porous cathodes with adequate pore sizes and morphologies to accommodate the expected solid discharge product volumes without losing pore interconnectivity, electronic percolation, or the film mechanical stability. Furthermore, interconnected porous cathode structures can promote the distribution of reactants over the entire cathode thickness rather than only at the electrode surface, thus facilitating the electrochemical reactions with lowered kinetic barriers. The homogeneous distribution of discharge products throughout the cathode, can greatly affect the battery performance such as cycle life, overpotential, and power.

It has been suggested that suitable pore template sizes resulting in high specific capacities are 35–100 nm for carbonized structures^18^,^19^,^20^, 200 nm for MWCNT^21^ and 250 nm for graphene^22^, respectively. However, the fundamental understanding of how pore size, shape, connectivity effects Li-oxygen battery performance is still poorly understood due to the lack of systematic control over porous structures and its subsequent effect on gas transport and electrochemistry. Here we use common microorganisms to directly template not only size but also pore shapes to create three dimensional, free-standing, and electrically conducting carbon nanotube-based films without the need for binders. Two different types of inexpensive, non-pathogenic bacteria are used to template either spherical (*S. epi*) or cylindrical (*E. coli*) macro-porous free-standing MWCNT films. To the best of our knowledge, this is the first demonstration of using such films as battery cathodes for 3D porous Li-oxygen batteries. Two general steps are involved in the direct templating method for production of porosity, (1) mixing of existing pore templates with the matrix material of interest, and (2) removal of templates to create pores^8^. For most applications, the ideal pore templates are compatible with the matrix material and easily removable without adversely affecting the functional properties of the matrix. By using bacteria as bio-templates, we were able to create macropores (diameter: 0.5–1 μm, length: 1–2 μm) in a solid matrix after removing the microorganisms by a mild chemical treatment (e.g. diluted bleach) and subsequent calcination (400 °C to remove cellular debris). This method proceeds without any significant damage to the matrix material (MWCNTs). Furthermore, these conditions are much milder than those used for conventional synthetic templates, which use strong acids and/or high temperature anneals. For example, silica-based porogens require hydrofluoric acid and thermal annealing temperatures of >800 °C for their removal^18^,^23^. We have used cheap and easily removable templating method to control both the porosity as well as pore inter-connectivity in MWCNT cathodes for Li-air batteries. The biomaterial-templated porous MWCNT films yielded a 30% improvement in the oxygen recovery efficiency compared to compact MWCNT films during the first battery cycle within the stable operating cell potential. Furthermore, by controlling the pore dimensions and pore interconnectivity of MWCNT films, we could enhance the power density by building an efficient percolation network for reactants to sustain the electrochemical reaction at high current densities (1.5–1.9 mA/cm^2^).

## Results

### Fabrication of porous MWCNT films with microorganisms

**Figure 1:**
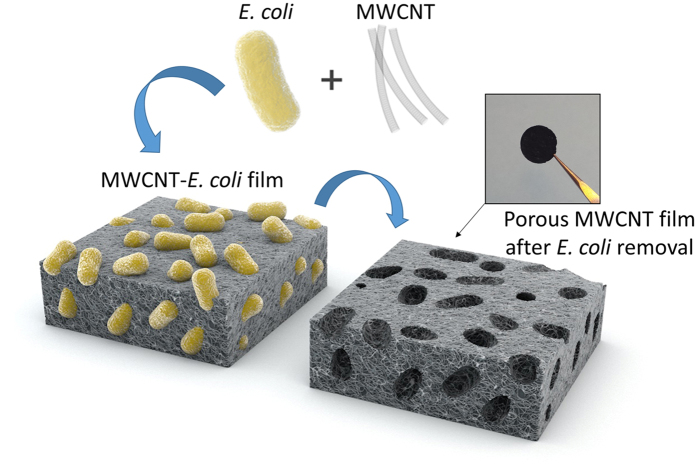
The schematic representation of the porous E-MWCNT film fabrication method, and an image of the final free-standing E-MWCNT film. E-MWCNT films are formed after the removal of biological pore templates, i.e., *E. coli*.

The porous MWCNT films were prepared in two simple steps; (1) filtering the mixture of microorganisms and MWCNTs to produce a film, and (2) removing the microorganisms, i.e., pore templates (Fig. 1) to create the macro-pores. Experimental details of the fabrication method are included in the methods section. Briefly, MWCNTs were first dispersed in deionized (DI) water in the presence of a surfactant using ultrasonication. The colloidal suspension of MWCNTs was added to the microorganism pore templating solution free of the growth media. Microorganism suspensions were used after their growth in a nutrient broth (tryptic soy broth, TSB). These bacterial suspensions were dialyzed against the DI water one day before mixing with the MWCNT dispersion to exchange the broth media with DI water. The mixture of the MWCNTs and microorganisms was vacuum filtered to form a free-standing film. Herein the films obtained right after vacuum filtration were called as ‘MWCNT-*E. coli*’ or ‘MWCNT-*S. epi*’ films, made from the mixture of MWCNT and *E. coli* or *S. epi*, respectively. In the second step, a protocol combining cell wall lysis with calcination to remove cell debris was developed to completely remove the microorganisms. Initially, we investigated several different cell lysis methods including incubating the microbes with strong oxidizing agents, reducing agents or surfactants. Specifically, the *E. coli* suspension in nutrient solution (~8.44 × 10^10^ cells in 200 μL) was mixed with base (NaOH 1 M, 600 μL), acid (H_2_SO_4_ 20 v/v % or HCl 15 v/v %, 600 μL), anionic surfactant (NaDBS, 2.5 w/v %, 600 μL), ethanol (100%, 600 μL), or bleach (10 v/v %, 600 μL) for 2 h. As a preliminary test, the efficiency of the microbial lysis was analyzed by comparing the optical micrographs of the cells in solution. The *E. coli* culture appeared most effectively decomposed by the bleach solution whereas NaOH, H_2_SO_4_, or ethanol yielded varying degrees of disruption of the microbial cell wall (Figure S1). The HCl and NaDBS treatments were not as effective as the other reagents in degrading the microorganisms under the conditions tested.

**Figure 2:**
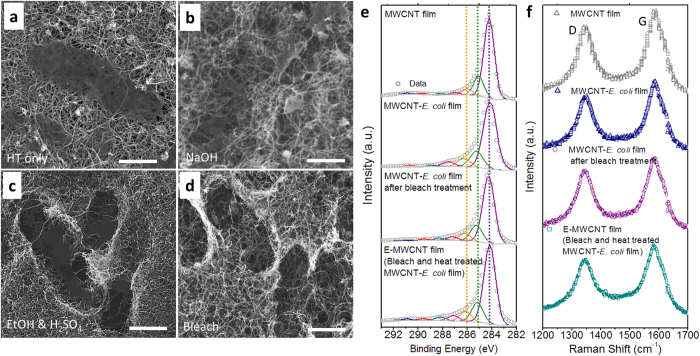
Comparison of the *E. coli* removal processes using different reagents and thermal treatment. (**a**–**d**) SEM images of porous MWCNT-*E. coli* films prepared by different removal protocols (1 μm length scale bar): (**a**) heat treatment only (HT, at 400 °C for 3 h under N_2_ flow), and after incubation with various reagents (**b**): NaOH (1 M, 1 h), (**c**): ethanol (100%, 2 h)/H_2_SO_4_ (20 v/v %, 1 h), (**d**): bleach (10 v/v %, 1 h)) followed by heat treatment. (**e**) XPS and (**f**) Raman spectra of porous MWCNT-*E. coli* films after each processing step.

The reagent-mediated cell lysis of the microorganisms embedded in MWCNT films disrupts the microorganism structure but leaves behind cell residue. For this reason, we combined chemical cell lysis with subsequent calcination to obtain MWCNT films that are largely free of biomolecular residues. Free standing MWCNT-*E. coli* films were incubated with the prescreened lysis reagents, followed by a heat treatment at 400 °C for 3 h under N_2_ flow to remove any residual cellular components. Microbial calcination has been used previously to denature membranes, ribosomes, and nucleic acids^24^. The decomposition of *E. coli* in MWCNT films was studied both by scanning electron microscopy (SEM) and energy dispersive X-ray spectroscopy (EDX) analyses (Fig. 2, Table S1). Thermal degradation without solvent-mediated cell lysis was not effective for complete microbial removal. For example, most of *E. coli* residue from cellular components remained in the MWCNT-*E. coli* film after only the thermal treatment. The presence of microbial residues were detected by EDX analysis showing that the atomic percentages of nitrogen (N) and oxygen (O)^25^ were higher in the heat-treated alone MWCNT-*E. coli* film (N: 6.2, O: 8.3, Table S1) as compared to MWCNT films alone (N: 0.8, O: 2.4). This validates the necessity for an initial reagent-mediated cell wall lysis step to facilitate the complete removal of the templates. Therefore, a combination of solvent treatment together with calcination was utilized for efficient biological porogen removal. MWCNT-*E. coli* films were first submerged in 400 mL of the different lysing reagents (Fig. 2b–d), followed by the thermal treatment. Among the selected chemicals, bleach showed the best results (Fig. 2d) for *E. coli* removal, as confirmed by EDX (Table S1). Because bleach contains a strong oxidizer, sodium hypochlorite (NaOCl), that can potentially oxidize the MWCNTs^26^,^27^, the incubation time of the MWCNT-*E. coli* films in the bleach solution was minimized (15 min) to effectively lyse the microbes while minimizing the oxidative damage to MWCNTs.

The carbon structure of MWCNTs was largely preserved during the *E. coli* removal process as confirmed by X-ray photoelectron spectroscopy (XPS, Fig. 2e for high resolution and Figure S2 for survey spectra) and Raman spectroscopy (Fig. 2f). Disruption of the periodic carbon structure of the MWCNTs was first determined by Raman spectroscopy, and detailed chemical bonding information was provided by XPS. In the Raman spectra, the D band (centered ~1342 cm^−1^) intensity is attributed to the disordered carbon materials in the CNTs while the G band (centered ~1581 cm^−1^) originates from in-plane tangential carbon-carbon stretching^28^. For this reason, the G band to D band peak intensity ratio (I_G_/I_D_) gives information about the amorphous content or the amount of defects in the CNT sample and thus can be used to monitor the change in defect levels from the optimal hexagonal carbon bonding structure^29^. After the free standing porous MWCNT film fabrication using *E. coli*, the I_G_/I_D_ ratio of E-MWCNT films remained similar to MWCNT films with no microorganisms added (Fig. 2f and Table 1). To estimate the extent of the formation of carbon defects, each XPS spectrum was analyzed by peak deconvolution with the fixed FWHM (full width at half maximum) using CASA XPS software. Fig. 2e shows C 1 s high resolution XPS spectra of MWCNTs (without *E. coli*), MWCNT-*E. coli*, bleach-treated MWCNT-*E. coli*, and MWCNT-*E. coli* films treated with both bleach and heat. The latter films are designated as E-MWCNT in this work (S-MWCNT represents bleach and heat treated MWCNT-*S. epi* films). Here we have grouped carbon bonding into two categories; the carbon bonds associated with (1) carbon-carbon (sp2 C = C at ~284.4 eV, sp3 C-C at ~285.5 eV), and (2) carbon-heteroatom bonds (C-OH, C=O, COOH C-N etc, at binding energies higher than 286 eV) to determine the disruption of carbon-carbon bonding in MWCNTs^30^,^31^. Although an increase in peak intensities (>286 eV) was not obvious in Fig. 2e, the carbon-based functional groups formed by the oxidation during the MWCNTs treatment process increased by approximately 8% (integrated C-C/C=C areas decreased from 88.07% to 80.4%, for the MWCNT and E-MWCNT samples, respectively) based on the peak deconvolution presented in Table 1.

**Figure 3:**
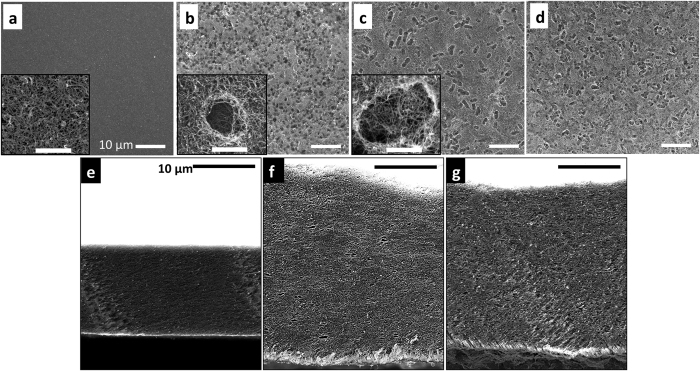
Top-down and cross-sectional SEM micrographs of MWCNT films (**a**,**e**), S-MWCNT films (**b**,**f**), and E-MWCNT films (**c**,**d**,**g**) with different *E. coli* initial cell numbers, (**c**) ~2.76 × 10^10^ and (**d**,**g**) ~8.19 × 10^10^ cells added during the film fabrication process. Scale bar for all figures represent 10 μm. The inset figures are high magnification images of pores with a 1 μm length scale bar. The flat planar cross sectional surfaces were prepared by ion milling to better visualize pore distribution of (**f**) the S-MWCNT (~1.28 × 10^11^ cells) and (**g**) E-MWCNT (~8.19 × 10^10^ cells) films, compared to MWCNT film (**e**). Because of the porosity, the S-MWCNT and E-MWCNT films were almost 1.8 times thicker than the MWCNT films, fabricated with the same amount of MWCNT.

Two parameters, pore shape and volume in the MWCNT films can be easily controlled by varying either the microorganism and/or the concentration without changing the fabrication process. Pore shape was changed by utilizing microorganisms with distinct shapes, e.g. cylindrical *E. coli* and spherical *S. epi*. While MWCNT films without microbes showed compact structures (Fig. 3a), microscale cylindrical or spherical shaped pores were formed in MWCNT films when microbes were employed as pore templates (Fig. 3b and c, respectively). The pore volume in the MWCNT film was controlled by adjusting the concentration of microorganism during the MWCNT film fabrication process. The initial microbial cell concentration estimate was obtained by measuring the optical density (OD at 600 nm) of the cell suspension with a spectrophotometer and converting it to viable cell concentrations using the quantitative spread plate method. In top-down SEM images presented in Fig. 3c and d, higher porosity was observed for the films made from larger number of cells (~8.19 × 10^10^ cells for Fig. 3d) compared to that made using a lower quantity (~2.76 × 10^10^ cells for Fig. 3c). In addition, the porous MWCNT films were thicker than MWCNT films without porogens (made with the same amount of MWCNT) due to the decreased densities of the porous samples. For example, the film thicknesses, as measured by cross-sectional SEM images of the macro-porous and compact MWCNT films, increased to ~38.5 μm (average value from Figs 3g and S3c) when ~8.19 × 10^10^ cells were used, from ~21.5 μm (average value from Figs 3e and S3a) when no microbes were used. The cross-sections of the MWCNT and E-MWCNT films (made with ~8.19 × 10^10^ cells) were prepared either by slope-cut ion milling (Fig. 3e–g) or snap fracture (Figure S3). At the same magnification, the MWCNT films showed a more compact cross-sectional structure (Fig. 3e) while the E-MWCNT films showed pores with average sizes ranging from 200–500 nm (Fig. 3f and g) to 1–2 μm. In addition, the E-MWCNT films were 80% thicker than MWCNT film. We also measured the effective pore volume (pore size <4 μm) by the mercury porosimetry. As expected, porous E-MWCNT films (~8.19 × 10^10^ cells) showed a higher volume of mercury intrusion (5.16 mL/g) than that of MWCNT films formed without microorganisms (3.76 mL/g) indicating a increased pore volume in the matrix (Figure S4). We note that MWCNT-*E. coli* films made with more than 1.41 × 10^11^ cells in the sample were mechanically unstable due to the large number of pore templates in the matrix.

### Li-oxygen battery performance improvement with porous MWCNT cathodes

**Figure 4:**
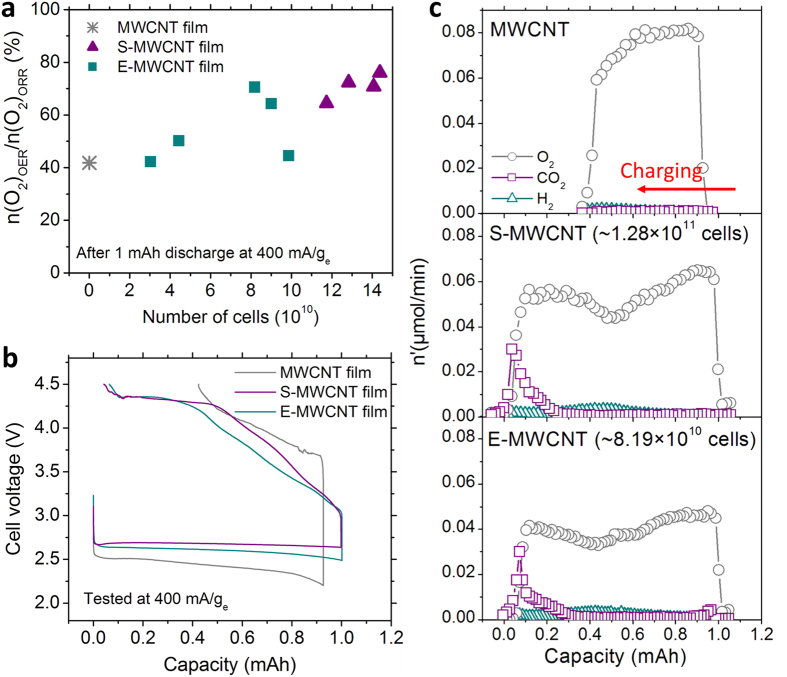
The Li-oxygen battery first cycle for MWCNT, S-MWCNT and E-MWCNT cathodes tested with 1 M LiTFSI in DME under 1.5 atm of O_2._ (**a**) Molar ratio between oxygen gas generated and consumed during the first cycle of the Li-oxygen battery at 400 mA/g_e_ at a fixed discharge capacity of 1 mAh (charging cut-off potential was 4.5 V vs. Li/Li^+^). (**b**) The first cycle voltage profiles that correspond to (**a**), MWCNT films, E-MWCNT films (~8.19 × 10^10^ *E. coli* cells) and S-MWCNT films (1.28 × 10^11^ *E. coli* cells). (**c**) Gas (O_2_, CO_2_, and H_2_) evolution rate profile during the first charging step of Li-oxygen battery corresponding to (**b**), measured by DEMS.

We evaluated the free-standing, binder-free porous MWCNT films made with microbes as Li-oxygen battery cathodes. Our results indicate that an improvement in the n(O_2_)_OER_/n(O_2_)_ORR_ efficiency (oxygen recovery ratio) of Li-oxygen battery can be achieved by adjusting the porosity using biological pore templates (Fig. 4a). Designing efficient porous cathode structures can significantly enhance the battery performance as Li-oxygen batteries operate by filling and subsequently emptying the available pores in the cathode with products formed during the discharge and charge processes. Cathode structures with three dimensionally interconnected pores can provide better access to electrochemical reaction sites throughout the entire thickness of the cathode even when some electrolyte channels are blocked. The molar amount of oxygen gas consumed during the discharge step (n(O_2_)_ORR_) was measured by tracking the pressure decay of the battery cells. Similarly, the oxygen gas generated during the charging step (n(O_2_)_OER_) was quantified by differential electrochemical mass spectrometry (DEMS) built in house^32^. The n(O_2_)_OER_/n(O_2_)_ORR_ efficiency was measured during the first cycle of Li-oxygen battery operation using 1 M LiTFSI (lithium bis(trifluoromethane sulfonyl)amide) in DME (1,2-dimethoxyethane)), at 400 mA/g_e_ (mass of cathode) under 1.5 atm O_2._ For the *E. coli* templated cathodes, the best n(O_2_)_OER_/n(O_2_)_ORR_ efficiency (71%) was obtained when the cell concentration used to generate the porosity was ~8.19 × 10^10^. For the spherical *S. epi* templated cathodes, ~5.6 times higher numbers of cells (~1.44 × 10^11^) were needed to achieve the highest efficiency (77%). The molar ratio between charge passed and oxygen consumed (e^−^/O_2_) during the discharge step for E-MWCNT, S-MWCNT, and MWCNT films was very close to two (Table 2), supporting the observation of Li_2_O_2_ formation during discharge as expected from the stoichiometry of Li_2_O_2_ formation reaction.

Both the *E. coli* and *S. epi* templated porous MWCNT films yielded much higher n(O_2_)_OER_/n(O_2_)_ORR_ efficiencies (>30%) compared to cathodes containing the same amount of MWCNTs but without the microbial templating. The average thicknesses of *E. coli* or *S. epi* templated films to achieve the optimum n(O_2_)_OER_/n(O_2_)_ORR_ values, were 38.5 μm (8.19 × 10^10^ cells) and 39 μm (1.28 × 10^11^ cells), respectively. As mentioned previously, these films were 80% thicker than the films prepared without microbes. Due to the size and stiffness differences between *E. coli* and *S. epi* templates, the number of cells added was different to achieve the optimum n(O_2_)_OER_/n(O_2_)_ORR_ values. However, both films had similar total porosity (i.e. density) as can be seen from the comparable final electrode thicknesses (Figs 3f,g and S3). During the charging process, E-MWCNT, S-MWCNT, and MWCNT films showed similar e^−^/O_2_ ratios (Table 2, ~2.6–2.7) indicating similar charge transfer efficiency for OER. Thus, the low oxygen recovery ratio of compact MWCNT cathodes (n(O_2_)_OER_/n(O_2_)_ORR_: 41.8%) most likely arises from the high charging overpotential that reaches the upper potential cut-off window (4.5 V) before it completely charges (Fig. 4b and c). In the “potential window” where electrochemical stability of the electrolyte is maintained (i.e. 2.2–4.5 V vs. Li/Li^+1^), S-MWCNT and E-MWCNT electrodes achieved 95.7 and 93.5% of coulombic efficiency, respectively, while the compact MWCNT electrodes reached only 54.3%. (Fig. 4b). The high overpotential of lower porosity cathodes could originate from the poor accessibility of electrochemical reaction sites throughout the cathode thickness. The thick insulating discharge products on the cathodes mostly accumulate either on the electrode-separator or electrode-air interface, where the concentration of reactants (Li^+^ and O_2_) is high. This can easily result in blockage of smaller pore entrances and lead to high resistance for the electrochemical reactions, requiring high charging overpotentials.

### The role of porosity in distributing discharge products

**Figure 5:**
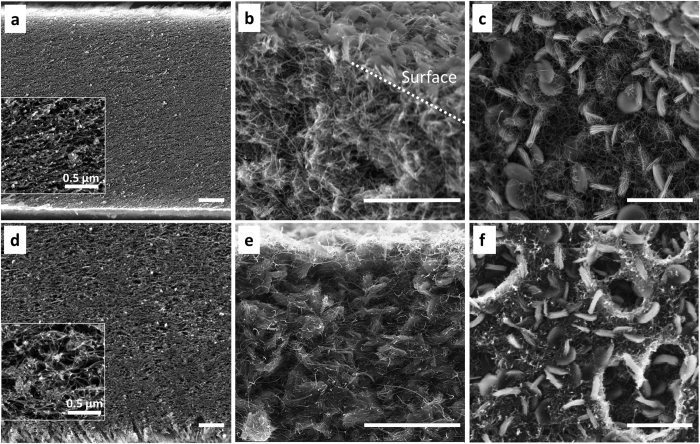
SEM images of MWCNT films (**a**–**c**) and E-MWCNT films (**d**–**f**) with the scale bars of 2 μm. (**a**,**d**) Cross-sectional views of the pristine states of MWCNT and E-MWCNT films, respectively. MWCNT (cross sectional view; b, top down view; **c**) and E-MWCNT (cross sectional view; e, top down view; **f**) cathodes discharged using 1 M LiTFSI DME containing 4,000 ppm H_2_O as the electrolyte.

**Figure 6:**
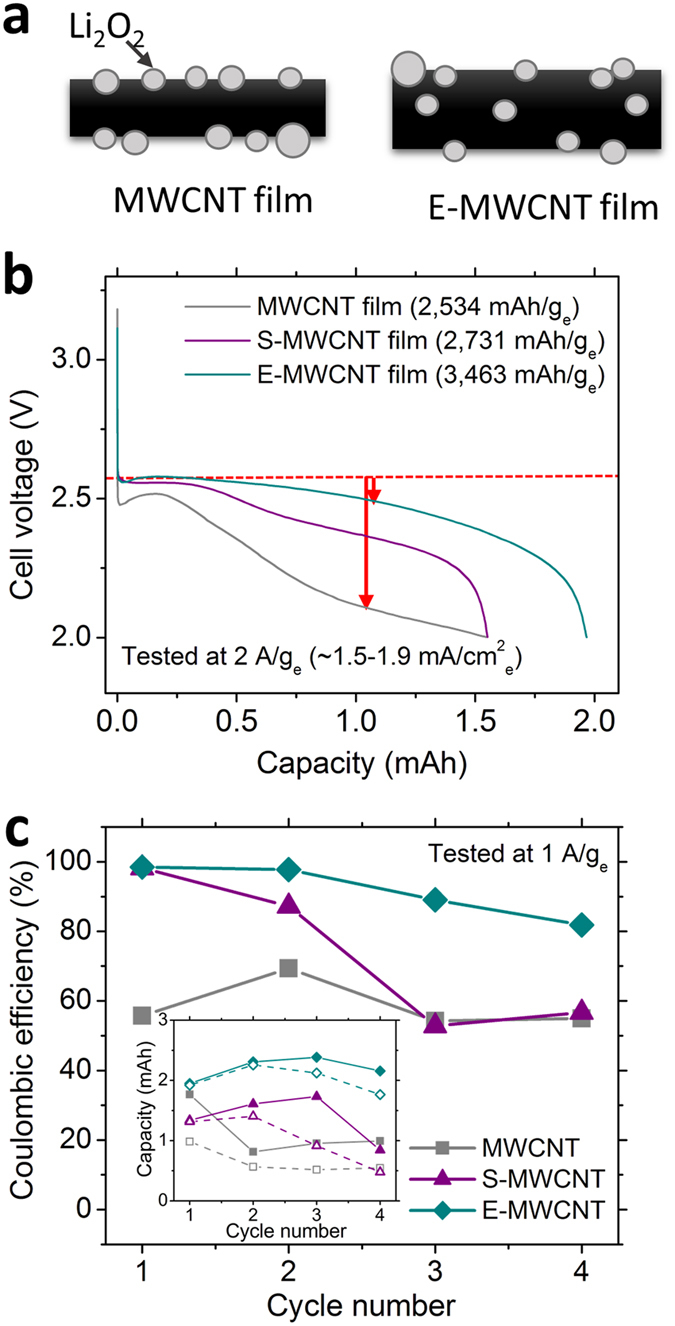
Li-oxygen battery performance using MWCNT, S-MWCNT and E-MWCNT cathode films, tested with 1 M LiTFSI in DME under 1.5 atm of O_2_. (**a**) The schematic showing the proposed distribution of discharge products in MWCNT and E-MWCNT cathodes. (**b**) The first full discharge capacity tested at 2 A/g_e_. Each cell was fully soaked in electrolyte for 1–2 h before the battery measurement. (**c**) Coulombic efficiencies of Li-oxygen battery with MWCNT, S-MWCNT and E-MWCNT cathodes for their first four deep cycles at 2–4.6 V of voltage windows tested at 1 A/g_e_. Inset: discharge (filled) and charge (empty) capacities of each cycles.

To investigate the effect of microscale cathode porosity on Li-oxygen battery operation, we probed the spatial distribution of the electrochemical reaction products by utilizing a visualization technique that allows the study of the products formed throughout the cathode using SEM. Previously, spatial analysis on Li-oxygen battery membranes or cathodes throughout their thickness has been studied using SEM^33^ or neutron scattering^34^, but the observation was unclear as the discharge products were grown as thin film form with nanoscale dimensions. Here, we intentionally induce toroidal Li_2_O_2_ growth during discharge within the cathode to provide a visual marker, during the discharge, by using electrolytes containing 4,000 ppm of H_2_O in the 1 M LiTFSI DME and testing against lithium iron phosphate (LiFePO_4_) anode^35^. The discharge product distribution within porous E-MWCNT cathodes compared to compact MWCNT cathodes was observed by cross-sectional SEM using microscopic toroidal Li_2_O_2_ growth for visualization. We see clearly that the toroidal Li_2_O_2_ growth takes place mainly at the bulk surface for a compact MWCNT electrode film (Fig. 5a–c). In contrast, highly porous E-MWCNT electrodes show a homogeneous distribution of toroidal Li_2_O_2_ deposits throughout the film after discharge (Fig. 5d–f). The cross-sectional SEM micrographs support the fact that the porosity increase resulted in more efficient utilization of the entire cathode, leading to a homogeneous distribution of electrochemical reaction sites (Fig. 6a) thus lowering overpotential upon charging. The uniform formation of embedded Li_2_O_2_ particles throughout the entire porous film thickness can be compared with the compact films where deposition is limited to the electrode surface.

### The power and cycle life improvement in Li-oxygen battery with porous MWCNT cathodes

We have successfully generated controlled porosity by using microorganisims as pore templates, thus yielding dramatic power improvements for MWCNT based Li-oxygen battery cathodes. The porous E-MWCNT cathodes achieved a 37% improvement in specific capacity at 2 A/g_e_ (corresponds to 1.5–1.9 mA/cm^2^) relative to that observed for a compact MWCNT (Fig. 6b, capacity of MWCNT cathodes: 1.55 mAh (2,534 mAh/g_e_ (corresponding energy density: 5,024 Wh/kg with 4,487 W/kg of power density)), S-MWCNT cathodes: 1.55 mAh (2,731 mAh/g_e_ (corresponding energy density: 6,695 Wh/kg with 4,869 W/kg of power density)), E-MWCNT cathodes: 1.97 mAh (3,463 mAh/g_e_ (corresponding energy density: 8,649 Wh/kg with 4,942 W/kg of power density))). This delivers a remarkable increase in the specific capacity of ~50% measured at 4 times higher current density compared to previous work where polystyrene beads were used as the pore templates for the Li-oxygen battery cathode fabrication^21^. Better utilization of porous electrodes not only provides better access to electrochemical reaction sites throughout the electrode when compared to the compact MWCNT cathodes, but also maintains “active” electrochemical reaction sites at high current densities. This is supported by the ~400 mV lower discharge overpotential for E-MWCNT films in Fig. 6b (red arrows) compared to the MWCNT cathodes without porogens. Although S-MWCNT cathodes improved both oxygen recovery ratio and coulombic efficiency compared to the MWCNT films, the power performance was similar to that obtained for MWCNT films. Because the film thickness increase was similar for the E-MWCNT and S-MWCNT films, the improved charging efficiency of the former suggests that not only the pore volume but also pore shape must play a pivotal role in improving battery performance. The higher power performance of E-MWCNT relative to S-MWCNT could be attributed to the better interconnectivity between pores generated from the anisotropic cylindrical shapes of *E. coli* compared to the small spherical shape of *S. epi* pore templates. (inset of Fig. 3b and c).

We have also investigated the preliminary Li-oxygen battery cycle life dependence on cathode porosity for MWCNT, S-MWCNT and E-MWCNT films within the fixed voltage window at 1 A/g_e_ (~0.84 mA/cm^2^, 2.0–4.6 V vs. Li/Li^+^, Fig. 6c and Figure S5). We cycled the Li-oxygen battery to 100% of depth of discharge rather than with the controlled, fixed discharge capacity to meaningfully evaluate their lifetimes. During the first four cycles of full discharge capacity, E-MWCNT cathodes maintained a coulombic efficiency ranging from 82–98.5%, while consolidated MWCNT electrodes showed poor cycling behavior with low coulombic efficiency (~55%, Fig. 6c). Due to the better charging capability of high porosity cathodes, the E-MWCNT cathodes demonstrated almost 2–3 times higher total discharge capacity (8.79 mAh) than MWCNT cathodes (4.53 mAh). This corresponds to a 94% improvement with E-MWCNT cathodes relative to the total discharge capacity achieved with the MWCNT cathodes maintained over the first four cycles. Although the S-MWCNT cathodes showed better coulombic efficiencies than MWCNT films in the first two cycles, these cathodes converged toward similar performance, after the third cycle. These results strongly suggest that increasing the porosity of cathodes can enhance the reversibility of a Li-oxygen battery as long as the interconnectivity of pores is maintained to maximize the utilization of cathode material. However, engineering and optimization of all cell components including incorporating electrolyte additives to improve the Li-oxygen battery cycle life remains as a future study with the broader scope for metal-air system.

In summary, bacteria-templated pore generation in Li-oxygen battery cathodes is reported for the first time. Furthermore, the dependence of Li-oxygen battery rechargeability performance on cathode porosity considering both the pore volume as well as pore shape has been demonstrated. Our results illustrate the importance of not only controlling porosity but also the interconnectivity of the pores within MWCNT cathode films. For MWCNT cathodes, cylindrical pores (shape anisotropy) were more beneficial than spherical shapes as evidenced by the improvement of the power and cycle life in a Li-oxygen battery. This is likely due to the better interconnectivity of cylindrical pores (derived from *E. coli* templates) in comparison with spherical pores because the volume required to reach percolation network is smaller for objects with increased shape anisotropy as it was shown in the literature^36^. The porous film fabrication with microbes offers lower material costs and more environmentally friendly processes as compared to using conventional sacrificial porogens. This method could, in principle, be further developed to create periodic pores by self-assembling biomolecules through genetic engineering of cell wall proteins. Furthermore, the decoration of inorganic materials on these pore templates could further enhance their applications into practical devices, such as solar cell, membrane, and sensors.

## Methods

### Fabrication of MWCNT-microorganisms (*E. coli* or *S. epi*) films

The bacterial suspension (*E. coli*: 2.3–5.6 × 10^9^ cells/mL and S. epi: 4.9–5.7 × 10^9 ^cells/mL in tryptic soy broth, TSB) was placed in a cellulose dialysis membrane (Sigma Aldrich, D9652-100FT) and dialyzed overnight in deionized (DI) water. Multi-walled carbon nanotube (MWCNT) powders (12 mg, >95%, OD 15 ± 5 nm, Length 5–20 μm, NanoLab Inc.) were dispersed with 0.48 mL (2.5% (w/v)) of sodium dodecylbenzene sulfonate (NaDBS, Sigma Aldrich) surfactant solution and 13.47 mL of DI water. The MWCNT dispersion was probe-sonicated (Branson, 102 C) in an ice bath for 40 min and the remaining MWCNT aggregates were collected by centrifugation (Beckman Coulter, Allegra X-22 Series Benchtop Centrifuge with SX4250 Swinging-bucket Rotor) at 4000 rpm (average 2177 rcf) for 20 min. The dispersed MWCNT and dialyzed microorganism solutions were mixed at room temperature for 2 h using a magnetic stirrer at 200 rpm. The MWCNT-microorganism solution was vacuum filtered using an anodisc membrane (Diameter. 47 mm, Pore diameter. 0.2 μm, Whatman). The filtered MWCNT-microorganism mixture, together with the filtration membrane, was initially immersed in 10 vol.% bleach (Clorox) solution for 15 min, and then rinsed overnight in DI water. The filtered MWCNT-microorganism mixture was separated from the membrane in DI water, and then freeze-dried (Labconco Freeze Dry System, Freezone^®^ 4.5) in order to preserve the 3D interconnected structure. The MWCNT-microorganism film was punched into 3/8″ diameter discs and heat-treated in a custom-made quartz tubing furnace at 400 °C for 3 h under 300 sccm N_2_ flow in order to remove the cell fragments. MWCNT films, containing no microorganisms, were fabricated as a control sample using the following process: the MWCNT dispersion (12 mg in 0.48 mL of NaDBS (2.5% (w/v)) solution) was prepared by the same method as previously described, using a probe sonicator and a centrifuge. The MWCNT dispersion was subsequently vacuum filtered (anodisc membrane, Dia. 47 mm, Pore Dia. 0.2 μm, Whatman). The filtered MWCNT dispersion formed a film that was easily separated from anodisc membrane. The MWCNT film was soaked overnight in DI water, freeze-dried and punched to produce 3/8″ diameter discs.

### Characterization of porous MWCNT-microorganisms (*E. coli* or *S. epi*) films

SEM imaging and EDX analysis were done using a FEI Helios Nanolab 400S field emission FIB/SEM with a Bruker Quantax 200 EDX detector. For the cross-sectional imaging, a 3 mm^2^ piece was cut from the sample with a razor blade and mounted on a titanium slope-cutting fixture, then air dried for 10 min. A cross-section was formed in a custom slope-cutting ion mill (repurposed from a Gatan PIPS Model 696), by Ar^+^ ion milling at 3.5 kV for 4 h. During milling, the sample temperature was kept constant at 100 °C. Carbon bonding states of the MWCNTs were determined by X-ray photoelectron spectroscopy (XPS, Physical Electronics Quantum 2000 ESCA Microprobe) using a monochromated Al Kα X-ray (hυ = 1,496 eV) source. 1,000 eV survey spectra (187.85 eV pass energy, 0.8 eV/step), and high resolution C 1 s and O 1 s spectra (46.95 eV pass energy, 0.2 eV/step) were recorded with charge neutralization, using a spot size of 200 μm. XPS spectra were corrected for charging by shifting the data with respect to the adventitious C peak located at 284.8 eV. Peak deconvolution was performed using the CASA XPS Software, with the fixed same full width at half maximum (FWHM) values for each peak. Raman spectra were collected using a Thermo Scientific DXR Raman Microscope (10 scans (20 s each) were acquired using a 532 nm (3.0 mW) laser under 50× objective lens).

### Electrochemical analysis

Prior to the electrochemical analysis, porous MWCNT cathodes and control samples (Diameter. 3/8″) were weighed, and dried overnight at 130 °C under vacuum. The Li-oxygen batteries were assembled using custom-made Swagelok^TM^ type cells inside an Ar-filled glove box (O_2_, H_2_O < 0.1 ppm, MBraun). Details of the custom-made cells are reported elsewhere^32^. The battery components were placed into the cell assembly in the following order: Li metal disc (Diameter. 11 mm, thickness 250 μm, FMC), one layer of Celgard^®^ separator (Diameter. 1/2″, 2500), porous MWCNT cathode (or MWCNT control sample) (Diameter. 3/8″), stainless steel mesh (Diameter. 12 mm), and stainless steel ring spacer (Height 1 mm). 65 μL of electrolyte (1 M LiTFSI (lithium bis(trifluoromethane sulfonyl)amide) in DME (1,2-dimethoxyethane), BASF Corp.) was then added and the cell was hermetically sealed in the Ar glove box. For water containing electrolytes, lithium iron phosphate (LiFePO_4_, MTI corporation, item number: bc-af-241lpf-ss, diameter: 12 mm) was used as the anode for Li-oxygen battery measurements. A multi-channel potentiostat (BioLogic, VMP3) and an in-house built differential electrochemical mass spectrometer (DEMS) were used to monitor the electrochemical performance and real-time *in situ* gas consumption/evolution analyses, respectively. The details of the operational principals of the DEMS are reported elsewhere^32^. After mounting to the DEMS set-up, the assembled battery was purged with O_2_ (^16^O_2_, ~1140 Torr, Research Purity, Matheson Tri-Gas^®^) through the inlet capillary and rested at open circuit voltage (OCV) for 30 min before the discharge. The type and amount of gas evolution was monitored by charging the battery under Ar environment (~1120 Torr, Research Purity, Matheson Tri-Gas^®^).

## Additional Information

**How to cite this article**: Oh, D. *et al*. Biotemplating pores with size and shape diversity for Li-oxygen Battery Cathodes. *Sci. Rep.* **7**, 45919; doi: 10.1038/srep45919 (2017).

**Publisher's note:** Springer Nature remains neutral with regard to jurisdictional claims in published maps and institutional affiliations.

## Supplementary Material

Supporting Information

## Figures and Tables

**Table 1:** Tabulation of relevant XPS data and Raman spectra in Fig. 2 for the MWCNT-*E. coli* film fabrication steps.

Sample	XPS	Raman		
	C=C & C-C Concentration (%)	G-band (cm^−1^)	D-band (cm^−1^)	I_G_/I_D_ ratio
MWCNT film	88.07	1586.88	1342.37	1.239
MWCNT-*E. coli* film	84.52	1586.46	1350.27	1.255
MWCNT-*E. coli* film after bleach treatment	83.51	1586.90	1346.79	1.203
E-MWCNT film (Bleach and heat treated MWCNT-*E. coli* film)	80.40	1581.32	1347.44	1.253

The data was obtained by integrating area concentration (%) of the deconvoluted peaks from XPS spectra in Fig. 2e. The G-band to D-band peak intensity ratio, and the positions of the bands from Raman spectra in Fig. 2f.

**Table 2:** Molar ratio between charge (e^−^) transferred and oxygen gas changes during discharge (ORR) and charge (OER) steps measured by DEMS.

Cathode	e^−^/O_2_	
	ORR	OER
MWCNT	2.09	2.71
S-MWCNT	2.06	2.73
E-MWCNT	1.98	2.62

The data was collected under the same conditions as Fig. 4b.
