# Erratum: Biotemplating pores with size and shape diversity for Li-oxygen Battery Cathodes

**DOI:** 10.1038/srep46808

**Published:** 2017-05-16

**Authors:** Dahyun Oh, Cagla Ozgit-Akgun, Esin Akca, Leslie E. Thompson, Loza F. Tadesse, Ho-Cheol Kim, Gökhan Demirci, Robert D. Miller, Hareem Maune

Scientific Reports 7: Article number: 45919; 10.1038/srep45919 published online: 04 04 2017; updated: 05 16 2017.

The original version of this Article contained an error in the spelling of the author Cagla Ozgit-Akgun, which was incorrectly given as Çagla Ozgit-Akgun. This error has now been corrected in the PDF and HTML versions of the Article.

