# Supplementary material for: Biotemplating pores with size and shape diversity for Li-oxygen Battery Cathodes
Source: Sci Rep. 2017 Apr 4;7:45919. doi: 10.1038/srep45919 (PMC5379672; doi:10.1038/srep45919)
Supplement: Supporting Information [file srep45919-s1.pdf]

Biotemplating pores with size and shape diversity for Li-oxygen Battery Cathodes

Dahyun Oh[1,], Çağla Ozgit-Akgün[2], Esin Akça[2], Leslie E. Thompson[1], Loza Tadesse[1, 3], Ho-Cheol Kim[1], Gokhan Demirci[2], Robert D. Miller[1,]*, Hareem Maune[1,]**

[1] IBM Almaden Research Center, San Jose, CA 95120, USA

[2]ASELSAN Inc. – Microelectronics, Guidance and Electro-Optics Business Sector, Ankara 06750, Turkey

[3] Minnesota State University Moorhead, Chemistry Department, Moorhead, MN 56563, USA

Estimated yield and cost calculation for bacteria production

(a) The price for 10 mL of *E. coli* (ATCC® 25922) is \$50.00 and 1,000 agar plates can be produced when 10 µL is used to make a single agar spread plate. One agar plate can produce roughly ~20 single colonies, 20 (colonies/plates) ×1000 (plates) = 20,000 colonies can be obtained for \$50.00, when the cost of nutrients, water and electricity is disregarded.

(b) Each colony can produce 10 mL of overnight stock which is sub-cultured in 100 mL of broth overnight to achieve an OD of 7.

(1) With one *E. coli* (ATCC® 25922, \$50.00) vial, we can get 110 mL/colony × 20,000 colonies ~2,200 L of OD ~7 bacterial cultures. If OD = 1 corresponds to 8.23×10^8 cells/mL of *E. coli*, then 8.23×10^8 cells/mL × 7 × 2200,000 mL/\$50.00 = <u>2.5×10^{14} cells/\$</u> is the approximate number generated per dollar.

(2) From Sigma-Aldrich, 15 mL of latex bead solution with mean particle diameters of 3 µm is available from \$455.10 (N, number of particles/mL = $1.828 \times 10^{11}/d^3$, d = particle diameter in µm). This corresponds to <u>2.23×10^8 beads/\$</u>.

Preparation of bacterial suspensions

(3) The bacteria were revived by rehydrating in 1 mL of tryptic soy broth (TSB) and then 5 mL of TSB was further added to grow it overnight at 37 °C while stirring at 250 rpm. Bacterial stocks were made from the 6 mL overnight growth by freezing the mixture of 0.6 mL of overnight stock with 0.4 mL of 50 % glycerol. 10 µl of frozen bacterial culture was used to make a streak TSB agar plate.

(4) To prepare the bacterial suspensions, 10 mL of overnight culture solution (16 h of growth at 37 °C with 250 rpm stirring) was prepared by inoculating one colony from a TSB agar plate. After diluting this overnight culture solution ten times with TSB broth, 100 mL of bacterial suspension was grown for another 12 h at 37 °C with 250 rpm stirring. Two cell types were used, (1) Serotype O6, Biotype 1 *E. coli* (ATCC® 25922) and (2) Staphylcoccus epidermidis (ATCC® 358914). The number of bacteria in solution was estimated by measuring the optical density (OD) of culture solution at 600 nm with UV spectroscopy and assuming that OD = 1 corresponds to 5×10^8 cells/mL for *E. coli*.

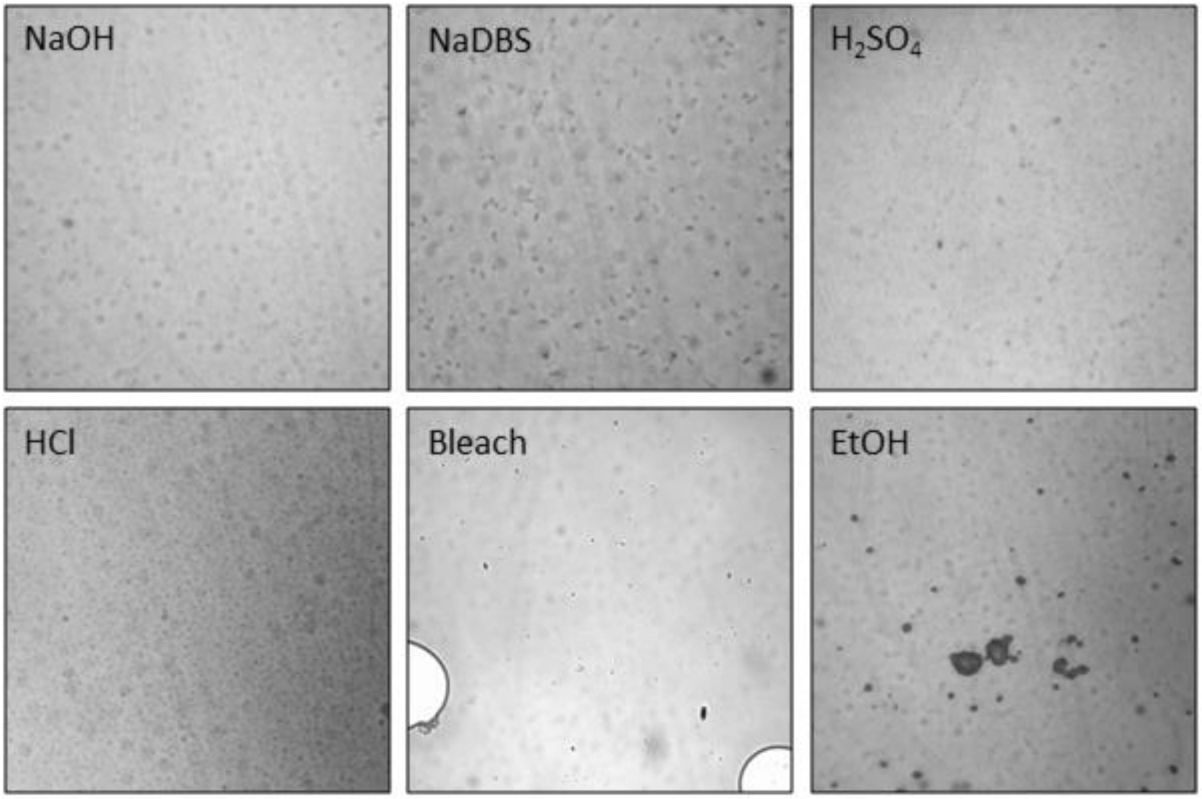

Figure S1. Optical microscopy images of *E.coli* solutions treated with different chemicals. The best result, in terms of removing *E. coli*, was obtained using bleach. Optical images were recorded with 50× objective lens.

Sample	C	N	O	Al	P	S	Fe	Ni
MWCNT film (without heat and bleach treatment)	96.0	0.8	2.4	0.3	0.0	0.2	0.3	0.0
MWCNT-*E.coli* film after heat treatment	84.6	6.2	8.3	0.0	0.4	0.2	0.3	0.0
E-MWCNT film (MWCNT-*E.coli* film after bleach and heat treatment side 1)	94.5	1.4	3.5	0.1	0.3	0.0	0.2	0.0
E-MWCNT film (MWCNT-*E.coli* film after bleach and heat treatment side 2)	94.9	1.4	3.0	0.0	0.2	0.1	0.3	0.1

Table S1. Atomic percentages (%) of the elements detected in MWCNT films measured by energy dispersive X-ray spectroscopy (EDX).

a

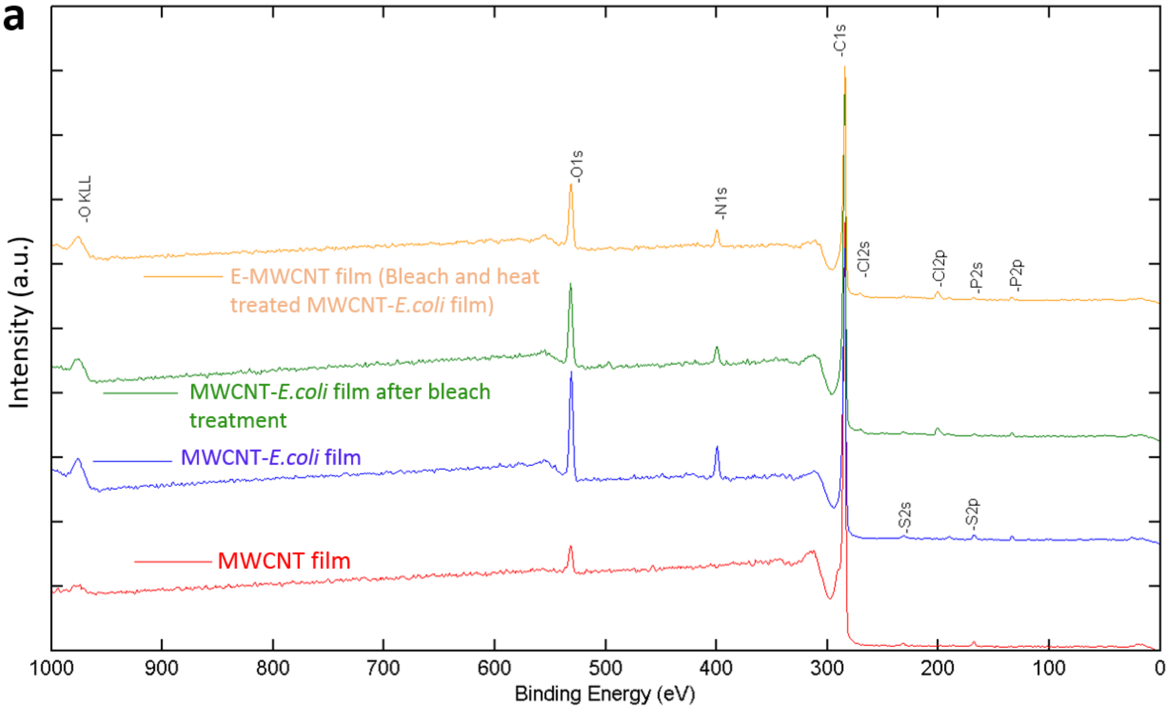

b

Sample	C1s	N1s	O1s	P2p	S2p	Cl2p
MWCNT film	96.4	0.0	3.2	0.0	0.4	0.0
MWCNT-*E.coli* film	80.5	5.2	13.3	0.5	0.5	0.0
MWCNT-*E.coli* film after bleach treatment	86.0	3.1	9.7	0.4	0.2	0.6
E-MWCNT film (Bleach and heat treated MWCNT-*E.coli* film)	83.8	3.5	11.0	0.5	0.3	0.9

Figure S2. The survey XPS spectra (a) and atomic percentage obtained from XPS spectrum (b) of MWCNT film, MWCNT-*E.coli* film, MWCNT-*E.coli* film after bleach treatment, and E-MWCNT film (Bleach and heat treated MWCNT-*E.coli* film).

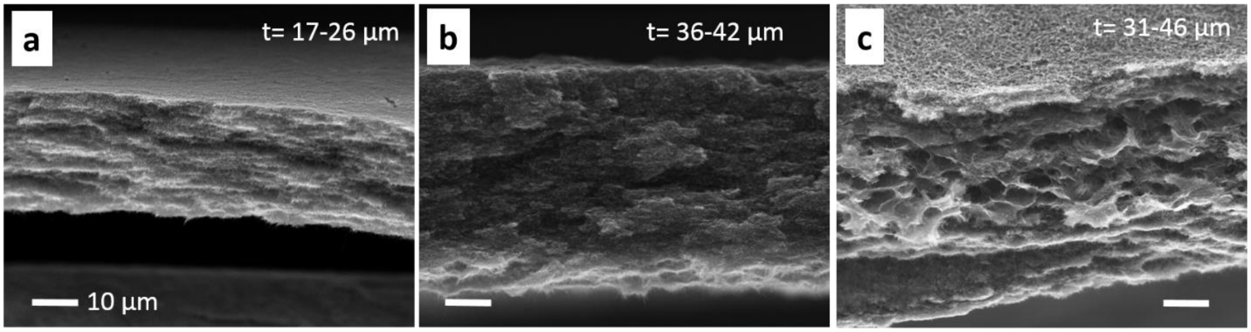

Figure S3. Cross-sectional SEM images of (a) MWCNT, (b) S-MWCNT, and (c) E-MWCNT films at the same magnification. The samples were prepared by mechanically breaking the films to obtain the cross-sectional view.

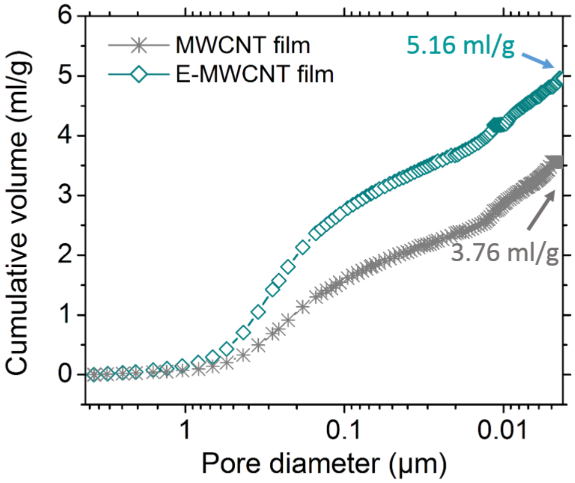

Figure S4. Mercury intrusion curve of MWCNT and E-MWCNT films (~8.19×10^{10} cells) for pores less than 4 µm (48-59,950 psia of pressure range).

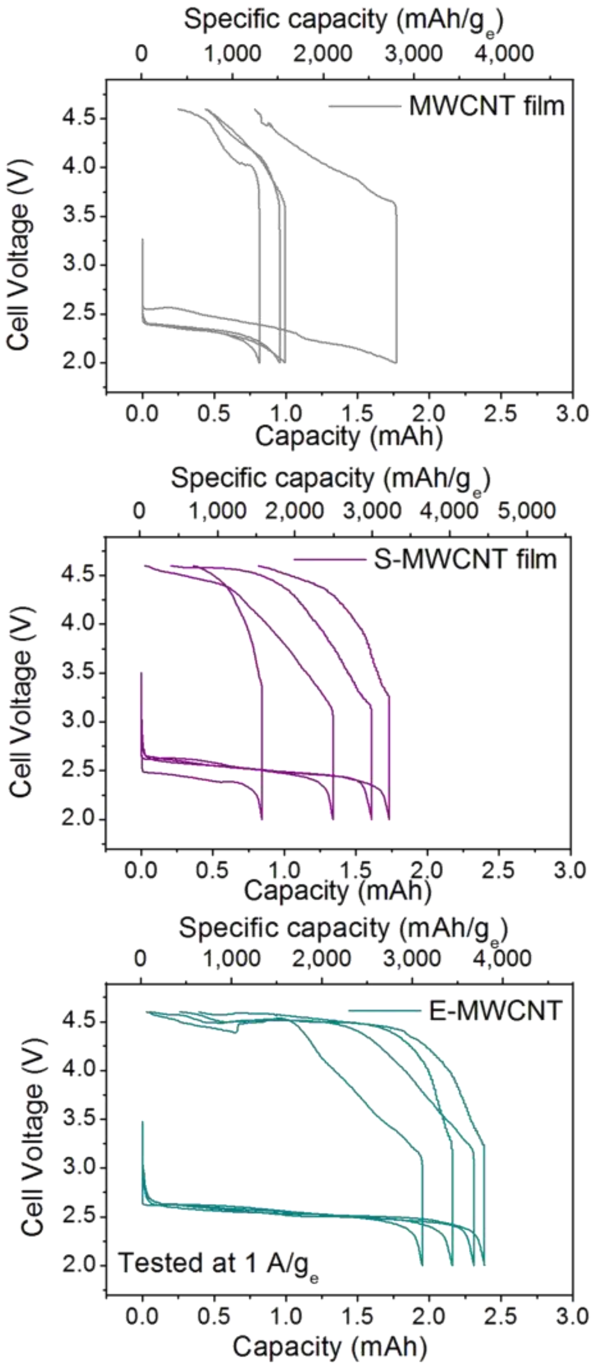

Figure S5. The cycling behaviors of MWCNT, S-MWCNT, and E-MWCNT films during the first four deep cycles with voltage windows of 2-4.6 V (Figure 6 in the manuscript). Li-oxygen batteries were tested at 1 A/g$_e$ of current density with 1 M LiTFSI in DME under 1.5 atm of O_2 .
